# The Homeostasis Model Assessment-insulin Resistance Index Is Inversely Associated with Serum Carotenoids in Non-diabetic Subjects

**DOI:** 10.2188/jea.16.71

**Published:** 2006-03-14

**Authors:** Minoru Sugiura, Mieko Nakamura, Yoshinori Ikoma, Masamichi Yano, Kazunori Ogawa, Hikaru Matsumoto, Masaya Kato, Makoto Ohshima, Akihiko Nagao

**Affiliations:** 1 Department of Citrus Research, National Institute of Fruit Tree Science.; 2 Department of Epidemiology, National Institute for Longevity Sciences.; 3 Food Materials Research Division, National Food Research Institute.

**Keywords:** Carotenoids, Diabetes Mellitus, Insulin Resistance, homeostasis model assessment-insulin resistance, Cross-Sectional Studies

## Abstract

**BACKGROUND:**

Carotenoids may reduce the risk for diabetes mellitus, but little is known about the association of insulin resistance with serum carotenoids in non-diabetic subjects. This study aimed to investigate whether the homeostasis model assessment-insulin resistance (HOMA-IR) index would be lower in the presence of high serum carotenoid concentrations in non-diabetic subjects.

**METHODS:**

A total of 812 subjects (256 males and 556 females) who had received health examinations in 2003 participated in the study. The associations of the serum-carotenoid concentrations and HOMA-IR were evaluated cross-sectionally. The multivariate-adjusted geometric means of HOMA-IR by the tertiles of the serum carotenoid concentration were calculated after adjusting for age, body mass index, systolic blood pressure, total cholesterol, triacylglycerols, current tobacco use, regular alcohol intake, exercise habits and total energy intake. Associations among high HOMA-IR (3.0+ mU×mmol/L^2^) across tertiles of serum carotenoid concentration were assessed by tests for logistic regression analysis.

**RESULTS:**

In male subjects, the multivariate adjusted geometric mean of HOMA-IR was inversely associated with the serum *β*-cryptoxanthin concentrations. In female subjects, an inverse association of the serum carotenoid concentration and HOMA-IR was observed in lycopene, *β*-cryptoxanthin, and zeaxanthin. The confounding factor-adjusted odds ratios (OR) for high HOMA-IR on the highest tertiles of serum *α*-carotene, *β*-carotene, *β*-cryptoxanthin, and zeaxanthin were 0.18 [95% confidence interval (CI): 0.06-0.52], 0.22 (95% CI: 0.07-0.67), 0.34 (95% CI: 0.12-0.96), and 0.30 (95% CI: 0.11-0.79), respectively, in male subjects. On the other hand, in female subjects, the adjusted OR for high HOMA-IR on the highest tertiles of serum lycopene and *β*-cryptoxanthin were 0.39 (95% CI: 0.21-0.73) and 0.51 (95% CI: 0.28-0.95), respectively.

**CONCLUSIONS:**

The serum antioxidant carotenoids were inversely associated with HOMA-estimated insulin resistance in non-diabetic subjects.

Antioxidant micronutrients, such as vitamins and carotenoids, exist in abundance in fruit and vegetables and have been known to contribute to the body’s defense against reactive oxygen species.^1^^,^^2^ Numerous epidemiologic studies have demonstrated that a high dietary consumption of fruit and vegetables rich in carotenoids or with high serum carotenoid concentrations results in lower risks of certain cancers and cardiovascular disease.^3^^-^^5^ Recent evidence has suggested that antioxidant vitamins and carotenoids may have a protective effect against diabetes mellitus.^6^^-^^13^

Insulin secretion from *β*-cells or insulin resistance plays an important role in the development of type-2 diabetes.^14^^,^^15^ Oxidative stress is thought to play a key role in the pathogenesis of type-2 diabetes mellitus by impairing insulin secretion or increasing insulin resistance.^16^^-^^19^ Therefore, antioxidant micronutrients, such as vitamins or carotenoids, would be expected to protect against the development of diabetes mellitus. Exogenous insulin secretion (*β*-cell function) is usually estimated with the oral glucose tolerance test. However, this method is unsuitable for epidemiologic studies because it requires a laborious procedure. On the other hand, many recent studies have reported that the homeostasis model assessment insulin resistance (HOMA-IR) index using fasting plasma glucose (FPG) and fasting serum insulin (endogenous insulin secretion) is a useful method for evaluating insulin resistance.^20^^-^^22^ Furthermore, Bonora et al. showed that the HOMA-estimated insulin resistance index is a better independent predictor of the incidence of type-2 diabetes than exogenous insulin secretion.^22^

This study aimed to investigate whether insulin resistance would be lower in the presence of a high serum carotenoid concentration in non-diabetic subjects. The associations of six serum carotenoid concentrations, i.e., lutein, lycopene, *α*-carotene, *β*-carotene, *β*-cryptoxanthin, and zeaxanthin, with the HOMA-estimated insulin resistance index were evaluated cross-sectionally.

## METHODS

Data used in the present study were derived from health examinations of residents, ranging in age from 30 to 70 years, of the town of Mikkabi, Shizuoka Prefecture, Japan, in April and May 2003. Mikkabi is located in western Shizuoka, and about 40% of its residents work in agriculture. Fruit trees are the key industry in Mikkabi, which is an important producer of Satsuma mandarin fruit in Japan. A total of 1,979 males and females were subjects for the health examination. As results, 1,448 participants (73.2% of total subjects) had received the health examination. Participants were recruited for this study, and informed consent was obtained from 886 subjects (302 males and 584 females). The response rate was 61.2%. This study was approved by the ethics committee of the National Institute of Fruit Tree Science and the Hamamatsu University School of Medicine.

Blood samples were obtained in the morning after overnight fasting. Serum was separated from blood cells by centrifugation and stored at -80°C until analysis of the serum carotenoid concentrations. The concentrations of six serum carotenoids, lutein, lycopene, *α*-carotene, *β*-carotene, *β*-cryptoxanthin, and zeaxanthin, were analyzed by reverse-phase high-performance liquid chromatography (HPLC) using *β*-apo-8’-carotenal as an internal standard at the laboratory of Public Health and Environmental Chemistry, Kyoto Biseibutsu Kenkyusho (Kyoto, Japan), as described previously.^23^

Serum total cholesterol and triacylglycerols were measured with an auto-analyzer using commercial kits (Determiner TC-II C for serum total cholesterol, Kyowa-Medics, Inc., Tokyo, Japan; Determiner TG-II C for serum triacylglycerols, Kyowa-Medics, Inc., Tokyo, Japan). Plasma samples were obtained in sampling vials containing sodium fluoride. FPG was measured with an auto-analyzer (Glucoroder MAX, SHINO-TEST, Inc., Tokyo, Japan). Fasting serum insulin was measured with an auto-analyzer (QUARTUS ImmunoCube, Kainos, Inc., Tokyo, Japan). All blood measurements, except for the serum carotenoid concentrations and fasting serum insulin, were conducted at the laboratory of Seirei Preventive Health Care Center (Shizuoka, Japan).

Height and body weight were measured by trained public health nurses. The body mass index (BMI) was calculated as the body weight (kg) divided by the height (m) squared. Blood pressure was measured using an automated sphygmomanometer Model BP-103iII (Nihon Colin, Inc., Aichi, Japan).

A self-administered questionnaire was used to collect information about the subjects’ history of diabetes and lifestyle, including tobacco use (current smoker, ex-smoker, or non-smoker), exercise (weekly participation), regular alcohol intake (6+ times per week) and dietary habits. Diet was assessed with a validated simple food-frequency questionnaire developed especially for the Japanese,^24^^,^^25^ and information about alcohol consumption and the daily intake of nutrients was obtained. The daily intakes of nutrients were estimated from the food intake frequencies per month with either standard portion size (for most of foods) or subject-specified usual portion size (for rice, breads, and alcoholic and non-alcoholic beverages). The total energy intake of all subjects was used in this report.

The estimate of the HOMA-IR score was calculated with the following formula: fasting serum insulin (mU/L) × FPG (mmol/L) / 22.5, as described by Bonora et al.^20^ Bonora et al. have reported that HOMA-estimated insulin resistance is an independent predictor of the incidence of diabetes^22^ and found that the incidence among those in the highest quartile of HOMA-IR was eightfold higher than that among those in the lowest quartile. In this study population, about 3.0 was the cut-off value between the highest and third quartile of HOMA-IR. Thus, a value equal to or more than 3.0 (mU×mmol/L^2^) of HOMA-IR was defined as high HOMA-IR in this data analysis.

For this study, we excluded subjects suffering from diabetes (7.0+ mmol/L of FPG) as defined by the American Diabetes Association diagnostic criteria.^26^ In addition, those who reported a history of diabetes in the self-administered questionnaire were excluded. Furthermore, one subject was excluded because of a fasting serum insulin level that was under the detection limit (0.4 mU/L), which made it impossible to obtain the HOMA-IR. A total of 812 non-diabetic subjects, subjects with normal fasting glucose (less than 6.1 mmol/L of FPG), and subjects with impaired fasting glucose (6.1-6.9 mmol/L of FPG) were included in further data analysis.

All subjects were categorized into four groups according to the quartile of HOMA-IR. Serum carotenoid concentrations, FPG, fasting serum insulin, HOMA-IR, and triacylglycerols were skewed toward the higher concentrations. These values were log_e_ (natural) transformed to improve the normality of their distribution. Analysis of covariance adjusted for age followed by Bonferroni multiple comparisons was used to test between-group differences in the HOMA-IR level. All variables were presented as an original scale.

The multivariate adjusted geometric mean of HOMA-IR by the tertiles of the serum carotenoid concentration was calculated after adjusting for age, BMI, systolic blood pressure, total cholesterol, triacylglycerols, current tobacco use, regular alcohol intake, exercise habits, and total energy intake using the analysis of covariance. Differences in the multivariate adjusted geometric mean of HOMA-IR among each tertile of serum carotenoid concentration were tested by Bonferroni multiple comparison. The serum carotenoid concentrations were assigned to three categories using the mean of the serum carotenoid concentrations in each tertile, and the associations among HOMA-IR across three categories were assessed with a test for linear trends using linear regression.

To assess the relationship between the serum carotenoid concentrations and high HOMA-IR, logistic regression analyses were performed after adjusting for age and BMI. Multivariable adjustment was further conducted to control potential confounders. Associations among high HOMA-IR (3.0+ mU×mmol/L^2^) across the tertiles of the serum carotenoid concentration were assessed by tests for logistic regression analysis. In the test for linear trends, the associations among high HOMA-IR across three categories assigned by means of the serum carotenoid concentration in each tertile were carried out by logistic regression analysis. We did not adjust each carotenoid concentration in the multivariate models because Pearson’s correlation analyses of serum carotenoid concentrations revealed significant positive correlations among all combinations of the six carotenoids.

The detection limit for the serum lycopene concentration for the method used in the study was 0.04 *μ*g/mL (0.075 *μ*mol/L), and the values below the limit of detection of the assay were marked as 0.03 *μ*g/mL (0.056 *μ*mol/L) in the analysis. All statistical analyses were performed using SPSS^®^ 12.0J for Windows (SPSS Inc., Chicago, IL, USA).

## RESULTS

Table 1 shows the characteristics of the study subjects stratified by HOMA-IR level. In male and female subjects, the BMI values in the third and highest quartiles of HOMA-IR were significantly higher than those in the lowest quartiles. In male subjects, diastolic blood pressure and serum total cholesterol in the highest quartiles of HOMA-IR were significantly higher than those in the lowest quartiles. Serum triacylglycerols in the second, third, and highest quartiles of HOMA-IR were significantly higher than those in the lowest quartile. Total energy intake and serum carotenoid levels were not different among the four groups stratified by HOMA-IR level. In contrast, in female subjects, systolic blood pressure and serum triacylglycerols in the third and highest quartiles of HOMA-IR were significantly higher than those in the lowest quartile. Diastolic blood pressure in the highest quartile of HOMA-IR was significantly higher than that in the lowest quartile. Although the total energy intake and serum carotenoid levels were not different among the four groups stratified by the HOMA-IR level, serum lycopene in the highest quartile of HOMA-IR was slightly lower, but not significantly, than that in the lowest quartile.

**Table 1.** Caracteristics of the study subject stratified by quartiles of the homeostasis model assessment-insulin resistance (HOMA-IR) index.

	Quartiles of the homeostasis model assessment-insulin resistance index										

	Lowest		Second			Third			Higest		
Male											
n	64		64			64			64		
Age (year)	55.8	(10.9)	55.7	(8.9)		56.0	(9.7)		57.3	(9.9)	
Body mass index (kg/m^2^)	21.7	(2.3)	22.6	(2.4)		23.6	(2.6)	***	25.4	(2.7)	***
Fasting plasma glucose (mmol/L)^§^	4.9	(4.8 - 5.0)	5.2	(5.1 - 5.4)	***	5.3	(5.2 - 5.4)	***	5.6	(5.5 - 5.7)	***
Fasting serum insulin (mU/L)^§^	3.9	(3.5 - 4.3)	7.2	(6.9 - 7.5)	***	10.4	(10.1 - 10.8)	***	14.8	(14.0 - 15.7)	***
HOMA-IR (mU × mmol / L2)^§^	0.8	(0.8 - 0.9)	1.7	(1.6 - 1.7)	***	2.5	(2.4 - 2.5)	***	3.7	(3.5 - 3.9)	***
Range		0.1 - 1.3		1.3 - 2.1			2.1 - 2.8			2.9 - 6.6	
Systolic blood pressure (mmHg)	129	(20.4)	128.4	(17.8)		129.7	(19.0)		137.9	(16.8)	
Diastolic blood pressure (mmHg)	77.8	(12.1)	78.5	(12.4)		79.7	(12.1)		84.8	(10.5)	**
Serum total cholesterol (mmol/L)	5.0	(0.8)	5.1	(0.9)		5.3	(0.9)		5.5	(0.8)	*
Serum triacylglycerols (mmol/L)^§^	1.0	(0.9 - 1.1)	1.2	(1.1 - 1.3)	*	1.2	(1.1 - 1.4)	**	1.5	(1.3 - 1.7)	***
Total energy intake (kcal/day)	2305.4	(516.5)	2236.2	(532.7)		2077.7	(531.5)		2128.3	(580.7)	
Serum carotenoid concentrations (mmol/L)^§^											
Lutein	0.52	(0.47 - 0.58)	0.57	(0.51 - 0.63)		0.55	(0.50 - 0.61)		0.51	(0.46 - 0.56)	
Lycopene	0.19	(0.15 - 0.22)	0.22	(0.18 - 0.27)		0.22	(0.18 - 0.27)		0.20	(0.17 - 0.24)	
*α*-Carotene	0.09	(0.08 - 0.11)	0.11	(0.09 - 0.13)		0.10	(0.09 - 0.12)		0.09	(0.08 - 0.10)	
*β*-Carotene	0.39	(0.33 - 0.45)	0.42	(0.35 - 0.49)		0.43	(0.37 - 0.49)		0.38	(0.33 - 0.44)	
*β*-Cryptoxanthn	1.09	(0.86 - 1.38)	0.95	(0.75 - 1.22)		1.14	(0.91 - 1.44)		1.08	(0.86 - 1.35)	
Zeaxanthin	0.23	(0.21 - 0.24)	0.23	(0.21 - 0.25)		0.24	(0.22 - 0.26)		0.23	(0.21 - 0.24)	
Current tobbaco use (%)	39.1		40.6			34.4			20.3		
Exercise habits (%)^†^	15.6		15.6			12.5			28.6		
Regular alcohol intake (%)^‡^	51.6		40.6			39.1			39.1		

Female											
n	139		139			139			139		
Age (y)	51.8	(10.1)	54.5	(9.7)		56.3	(9.7)		56.3	(9.9)	
Body mass index (kg/m2)	21.4	(2.3)	21.8	(2.6)		22.7	(3.0)	***	24.5	(3.7)	***
Fasting plasma glucose (mmol/L)^§^	4.8	(4.8 - 4.9)	5.0	(5.0 - 5.1)	***	5.2	(5.1 - 5.2)	***	5.5	(5.4 - 5.5)	***
Fasting serum insulin (mU/L)^§^	4.7	(4.4 - 4.9)	7.6	(7.5 - 7.8)	***	10.1	(9.9 - 10.3)	***	15.4	(14.8 - 16.0)	***
HOMA-IR (mU × mmol / L2)^§^	1.0	(0.9 - 1.1)	1.7	(1.7 - 1.7)	***	2.3	(2.3 - 2.4)	***	3.7	(3.6 - 3.9)	***
Range		0.2 - 1.4		1.4 - 2.0			2.0 - 2.7			2.8 - 9.9	
Systolic blood pressure (mmHg)	122.6	(19.0)	125.7	(17.3)		131.7	(18.3)	*	136.1	(19.5)	***
Diastolic blood pressure (mmHg)	72.2	(11.0)	74.2	(9.9)		76.7	(10.8)		78.8	(11.6)	***
Serum total cholesterol (mmol/L)	5.4	(0.9)	5.5	(0.9)		5.7	(0.8)		5.7	(0.9)	
Serum triacylglycerols (mmol/L)^§^	0.8	(0.7 - 0.8)	0.9	(0.8 - 0.9)		1.0	(0.9 - 1.1)	**	1.1	(1.0 - 1.2)	***
Total energy intake (kcal/day)	2008.3	(641.2)	2030.5	(545.0)		2054.3	(670.2)		2063.5	(518.2)	
Serum carotenoid concentrations (mmol/L)^§^											
Lutein	0.58	(0.54 - 0.62)	0.61	(0.58 - 0.65)		0.59	(0.55 - 0.63)		0.55	(0.52 - 0.58)	
Lycopene	0.32	(0.29 - 0.36)	0.35	(0.31 - 0.39)		0.27	(0.24 - 0.30)		0.25	(0.22 - 0.27)	
*α*-Carotene	0.14	(0.13 - 0.15)	0.15	(0.14 - 0.16)		0.14	(0.13 - 0.15)		0.13	(0.12 - 0.14)	
*β*-Carotene	0.70	(0.64 - 0.77)	0.77	(0.71 - 0.83)		0.72	(0.66 - 0.79)		0.64	(0.59 - 0.69)	
*β*-Cryptoxanthn	1.43	(1.23 - 1.67)	1.61	(1.41 - 1.84)		1.64	(1.44 - 1.87)		1.42	(1.25 - 1.61)	
Zeaxanthin	0.24	(0.22 - 0.25)	0.25	(0.24 - 0.26)		0.23	(0.22 - 0.24)		0.23	(0.22 - 0.24)	
Current tobbaco use (%)	1.4		1.4			0.7			0.0		
Exercise habits (%)^†^	16.5		25.2			26.8			27.7		
Regular alcohol intake (%)^‡^	2.9		2.2			1.4			1.4		

Data are mean (standard deviation), geometric mean (95% confidence interval), range, or percent.

†: 1+ times/week

‡: 6+ times/week

* : P<0.05, **: P<0.01 and ***: P<0.001 vs lowest quartile of HOMA-IR by analysis of covariance adjusted for age followed by Bonferroni multiple comparison test

§: These variables were represented as original scale after analysis by log (natural) transformed values

**Figure 1.**
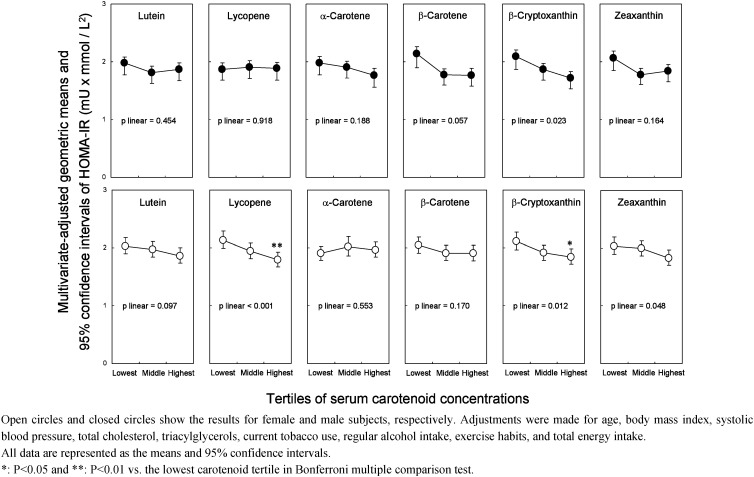
Multivariate adjusted geometric means of homeostasis model assessment-insulin resistance (HOMA-IR) by tertiles of the serum carotenoid concentrations.

The multivariate adjusted geometric means of HOMA-IR associated with the tertiles of each serum carotenoid concentration are shown in Figure 1. In male subjects, although no significant differences among each tertile were observed in all six serum carotenoids, the multivariate adjusted geometric means of HOMA-IR showed a significant decreasing trend under linearity with the tertiles of serum *β*-cryptoxanthin. On the other hand, in female subjects, the multivariate adjusted geometric means of HOMA-IR in the highest tertiles of serum lycopene and *β*-cryptoxanthin were significantly lower than those in the lowest tertiles.

The odds ratios of high HOMA-IR (3.0+ mU×mmol/L^2^) associated with the tertiles of six serum carotenoid concentrations after adjustments for confounding factors are shown in Table 2. After adjustments for age and BMI, a significantly lower odds ratio for high HOMA-IR in male subjects was observed in the highest tertiles of serum *α*-carotene compared to the respective lowest tertile used for reference. In female subjects, a significantly lower odds ratio for high HOMA-IR was observed in the highest tertiles of serum lycopene compared to the respective lowest tertile used for reference. Multivariate adjustment was further conducted to control potential confounding factors. After adjustments for systolic blood pressure, total cholesterol, triacylglycerols, current tobacco use, regular alcohol intake, exercise habits, and total energy intake in male subjects, a significantly lower odds ratio for high HOMA-IR was observed in the highest tertiles of serum *α*-carotene, *β*-carotene, *β*-cryptoxanthin, and zeaxanthin compared to the respective lowest tertile used for reference. In the female subjects, a significantly lower multivariate adjusted odds ratio for high HOMA-IR was observed in the highest tertiles of serum lycopene and *β*-cryptoxanthin compared to the respective lowest tertile used for reference.

**Table 2.** Odds ratios (ORs) of tertiles of serum carotenoid concentrations on high homeostasis model assessment-insulin resistance.

Serum carotenoid concentrations		n	Range (*μ*mol/L)	Model 1^†^			Model 2^‡^		
	
				OR	95% CI	P for trend	OR	95% CI	P for trend
Male									
Lutein	Lowest	88	0.16 - 0.46	1.00	(reference)	0.632	1.00	(reference)	0.283
	Middle	82	0.47 - 0.62	0.89	(0.40 - 1.98)		0.71	(0.29 - 1.74)	
	Highest	86	0.63 - 1.85	0.82	(0.35 - 1.88)		0.61	(0.24 - 1.54)	

Lycopene	Lowest	91	0.06 - 0.15	1.00	(reference)	0.835	1.00	(reference)	0.476
	Middle	79	0.17 - 0.30	1.23	(0.55 - 2.75)		1.08	(0.45 - 2.61)	
	Highest	86	0.32 - 1.06	1.06	(0.46 - 2.47)		0.69	(0.27 - 1.77)	

*α-*Carotene	Lowest	88	0.04 - 0.07	1.00	(reference)	0.041	1.00	(reference)	0.002
	Middle	91	0.09 - 0.11	0.60	(0.28 - 1.32)		0.43	(0.18 - 1.02)	
	Highest	77	0.13 - 0.73	0.40	(0.16 - 0.96)		0.18	(0.06 - 0.52)	

*β-*Carotene	Lowest	85	0.07 - 0.30	1.00	(reference)	0.071	1.00	(reference)	0.009
	Middle	87	0.32 - 0.50	0.55	(0.24 - 1.26)		0.41	(0.16 - 1.06)	
	Highest	84	0.52 - 1.96	0.44	(0.18 - 1.06)		0.22	(0.07 - 0.67)	

*β-*Cryptoxanthin	Lowest	86	0.13 - 0.72	1.00	(reference)	0.170	1.00	(reference)	0.045
	Middle	85	0.76 - 1.65	0.47	(0.20 - 1.11)		0.33	(0.12 - 0.88)	
	Highest	85	1.66 - 8.19	0.55	(0.23 - 1.30)		0.34	(0.12 - 0.96)	

Zeaxanthin	Lowest	80	0.09 - 0.19	1.00	(reference)	0.185	1.00	(reference)	0.015
	Middle	89	0.21 - 0.25	0.66	(0.29 - 1.47)		0.50	(0.21 - 1.22)	
	Highest	87	0.26 - 0.54	0.57	(0.25 - 1.31)		0.30	(0.11 - 0.79)	

Female									
Lutein	Lowest	189	0.19 - 0.49	1.00	(reference)	0.613	1.00	(reference)	0.372
	Middle	182	0.51 - 0.67	1.40	(0.82 - 2.40)		1.35	(0.77 - 2.36)	
	Highest	185	0.69 - 1.90	0.86	(0.48 - 1.53)		0.76	(0.41 - 1.40)	

Lycopene	Lowest	192	0.06 - 0.22	1.00	(reference)	0.014	1.00	(reference)	0.005
	Middle	175	0.24 - 0.41	0.95	(0.57 - 1.59)		0.84	(0.49 - 1.44)	
	Highest	189	0.43 - 1.38	0.45	(0.25 - 0.81)		0.39	(0.21 - 0.73)	

*α-*Carotene	Lowest	224	0.06 - 0.11	1.00	(reference)	0.240	1.00	(reference)	0.359
	Middle	125	0.13 - 0.15	1.50	(0.83 - 2.71)		1.54	(0.83 - 2.86)	
	Highest	207	0.17 - 0.89	1.38	(0.82 - 2.34)		1.33	(0.76 - 2.31)	

*β-*Carotene	Lowest	191	0.13 - 0.56	1.00	(reference)	0.210	1.00	(reference)	0.125
	Middle	184	0.58 - 0.89	0.81	(0.47 - 1.38)		0.77	(0.44 - 1.33)	
	Highest	181	0.91 - 2.63	0.70	(0.39 - 1.23)		0.63	(0.34 - 1.15)	

*β-*Cryptoxanthin	Lowest	185	0.16 - 1.09	1.00	(reference)	0.105	1.00	(reference)	0.034
	Middle	186	1.10 - 2.35	0.79	(0.45 - 1.38)		0.68	(0.38 - 1.23)	
	Highest	185	2.37 - 8.61	0.61	(0.34 - 1.11)		0.51	(0.28 - 0.95)	

Zeaxanthin	Lowest	168	0.11 - 0.19	1.00	(reference)	0.766	1.00	(reference)	0.286
	Middle	208	0.21 - 0.26	1.37	(0.80 - 2.35)		1.36	(0.78 - 2.38)	
	Highest	180	0.28 - 0.62	0.93	(0.51 - 1.66)		0.72	(0.38 - 1.35)	

CI: confidence interval

†: Age and body mass index were adjusted.

‡: Age, body mass index, systolic blood pressure, total cholesterol, triacylglycerols, current tobacco use, regular alcohol intake, exercise habits, and total energy intake were adujsted

## DISCUSSION

This study aimed to investigate whether insulin resistance would be lower in the presence of a high serum carotenoid concentration in non-diabetic subjects. The results indicate that HOMA-estimated insulin resistance is inversely associated with serum *α*-carotene, *β*-carotene, *β*-cryptoxanthin, and zeaxanthin in male subjects and inversely associates with serum lycopene and *β*-cryptoxanthin in female subjects. Insulin resistance plays an important role in the development of type-2 diabetes.^14^^,^^15^ Thus, our findings further support the hypothesis that antioxidant carotenoids may have a protective effect against the development of diabetes mellitus.

Insulin resistance is currently being measured using the glucose clamp technique.^27^^-^^30^ This method is highly sensitive and shows high reproducibility; however, it is laborious and expensive and, therefore, unsuitable for large-scale or epidemiologic studies. Many recent studies have reported that the HOMA-estimated insulin resistance index using FPG and fasting serum insulin is a useful method for evaluating insulin resistance.^20^^-^^22^ Very recently, Bonora et al. have reported that HOMA-estimated insulin resistance was an independent predictor of the incidence of diabetes.^22^ In this report, they indicated that the incidence among those in the highest quartile of HOMA-IR was eightfold higher than that among those in the lowest quartile. Therefore, we used the HOMA-IR index to evaluate the association of serum carotenoids and insulin resistance in this survey. Our results are the first to indicate the inverse associations of serum carotenoid concentrations with HOMA-IR in non-diabetic subjects.

Previously, Ford et al. reported the cross-sectional associations between serum carotenoids and insulin concentrations from the population-based US Third National Health and Nutrition Examination Survey.^10^ In this report, they found that serum *α*-carotene, *β*-carotene, lycopene, *β*-cryptoxanthin, and lutein/zeaxanthin were inversely correlated with fasting serum insulin concentration. In addition, in a small cross-sectional study, Facchini et al. reported an inverse correlation between serum *α*-carotene, *β*-carotene, and lutein with insulin resistance.^31^ However, these two studies did not evaluate the associations of serum carotenoids and insulin concentrations among non-diabetic subjects. It is not known whether insulin resistance would be lower in the presence of high serum carotenoid concentrations in non-diabetic subjects. Recently, Ylonen et al. reported an inverse association of serum *β*-carotene concentration with insulin resistance in subjects with a family history of type-2 diabetes.^32^ In this report, the proportion of subjects with normal fasting glucose status was approximately 79% among the study population. In contrast, in our study subjects, most of the males (91.0%) and females (95.5%) had normal fasting glucose. Therefore, it is possible that serum carotenoids are inversely associated with insulin resistance within healthy non-diabetic subjects. In fact, similar results were observed even after excluding subjects who had impaired fasting glucose (data not shown).

One recent cohort study about the associations of antioxidants intake with the incidence of type 2 diabetes has been reported by Montonen et al.^13^ In this study, they found that, among single carotenoids, dietary *β*-cryptoxanthin intake was significantly associated with a reduced risk of type 2 diabetes. A significant inverse association of serum *β*-cryptoxanthin with HOMA-IR was observed in both male and female subjects in our study. These results suggest that the development of type-2 diabetes may be reduced by the dietary intake of *β*-cryptoxanthin. The role of antioxidant carotenoids in the pathogenesis of insulin resistance is uncertain. It has been reported that oxidative stress impairs insulin action.^33^ Furthermore, a significant relationship between fasting plasma free radical production and insulin action in non-insulin-dependent diabetes has been reported.^34^ Therefore, antioxidant carotenoids may have a protective effect against oxidative stress in the pathogenesis of insulin resistance.

In this study, a significant inverse association of serum *β*-cryptoxanthin concentration with HOMA-IR was noted in male and female subjects. Furthermore, similar inverse associations of serum lutein, *β*-carotene, and zeaxanthin with HOMA-IR were observed in male and female subjects. In contrast, discrepant results about the associations of serum lycopene and *α*-carotene concentrations with HOMA-IR were observed between male and female subjects. We have no clear explanation for these differences. We concluded that an interaction between sex and carotenoid with insulin resistance is possible or that the sex differences observed occurred by chance. One possible explanation is that alcohol consumption may influence the association of serum carotenoid with insulin resistance. Light-to-moderate alcohol consumption is well known to enhance insulin sensitivity.^35^^-^^37^ Furthermore, in our study population, a considerably lower level of serum *α*-carotene compared with other carotenoids, such as *β*-cryptoxanthin or *β*-carotene, might be one of the causes for the discrepancies. The cause of the discrepant results between male and female subjects in the present study should be clarified in further studies.

There are two limitations in the present study. First, the data obtained here consisted of cross-sectional analyses. Therefore, our data limits inferences on temporality and causation. Secondly, we have no data about the serum levels of other antioxidant vitamins, such as vitamin C and *α*-tocopherol.

In conclusion, serum carotenoid concentrations, especially in *β*-cryptoxanthin, were inversely associated with HOMA-estimated insulin resistance. These inverse associations suggest that these carotenoids may act as suppressors against insulin resistance. To determine whether serum antioxidant carotenoids are effective in the prevention of insulin resistance and type-2 diabetes, further cohort studies or intervention studies will be required.
